# Population-based risk adjusted outcomes for out-of-hospital cardiac arrest

**DOI:** 10.1038/s44325-026-00108-7

**Published:** 2026-03-02

**Authors:** Ethan E. Abbott, David G. Buckler, Kevin Petrozzo, Douglas J. Wiebe, Benjamin S. Abella, Brendan G Carr, Alexis M. Zebrowski

**Affiliations:** 1 Department of Emergency Medicine, Icahn School of Medicine at Mount Sinai, New York, NY USA; 2 Department of Population Health Science and Policy, Icahn School of Medicine at Mount Sinai, New York, NY USA; 3 Windreich Department of Artificial Intelligence and Human Health, Icahn School of Medicine at Mount Sinai, New York, NY USA; 4 Charles Bronfman Institute for Personalized Medicine, Icahn School of Medicine at Mount Sinai, New York, NY USA; 5 The Hasso Plattner for Digital Health at Mount Sinai Icahn School of Medicine at Mount Sinai, New York, NY USA; 6 Institute for Health Equity Research, Icahn School of Medicine at Mount Sinai, New York, NY USA; 7 Center for Resuscitation Science and Innovation, Department of Emergency Medicine, Icahn School of Medicine at Mount Sinai, New York, USA; 8 Department of Epidemiology, School of Public Health, University of Michigan, Ann Arbor, MI USA

**Keywords:** Myocardial infarction, Ventricular fibrillation

## Abstract

Out-of-hospital cardiac arrest (OHCA) impacts public health, with variable survival across the US. This study used a population-based risk adjustment model to understand factors influencing regional variability in OHCA survival to hospital discharge. We evaluated 202,406 OHCA cases from 2013-2015 Medicare Fee-For-Service claims across 205 hospital regions. A matched cohort from the Cardiac Arrest Registry to Enhance Survival (CARES) and Medicare claims was used to develop logistic regression models predicting survival. Standardized Incidence Ratios (SIRs) identified regions performing better or worse than expected. Of 205 regions, 101 (49.3%) demonstrated lower-than-expected risk-adjusted survival, while only 9 (4.4%) had higher-than-expected survival. Overperforming regions had smaller populations, higher proportions of residents aged 65 + , and more large hospitals (400+ beds). Hospitals with ≥100 beds were more likely in overperforming regions, while cardiac catheterization capability showed inverse association. These nationwide disparities highlight the need for targeted interventions and regionalized care approaches to improve survival rates.

## Introduction

An estimated 400,000 out-of-hospital cardiac arrests (OHCA) occur each year in the US, with generally low rates of survival to discharge and significant regional variability in survival across communities^1–4^. Since 2010, the American Heart Association (AHA) has advocated for the development of regionalized resuscitation systems of care for OHCA to align with the improved systems-based outcomes seen with other time-sensitive conditions, such as stroke, ST-elevation myocardial infarction (STEMI), and trauma^5^. Regionalized resuscitation systems of care for OHCA include cardiac resuscitation centers (CRC), which are composed of multiple participants linked together to include communities, hospitals, and EMS systems across a given designated geographic area^6^.

Emergency care-sensitive conditions (ECSC), described by Carr et al.^7^, represent conditions, such as trauma, STEMI, stroke, and OHCA and require a system of care that can respond effectively and rapidly to meet the unique time-sensitive needs of these conditions. Regionalized systems of care have been demonstrated to be an important factor in improved outcomes for multiple ECSC’s^8–11^. However, the process of building coordinated regionalized resuscitation systems has been hindered by a lack of transparent outcomes and population-based incentives, as well as current models of care that do not utilize shared accountability between individual hospitals and healthcare systems. Fragmented EMS and hospital networks, variability in interfacility transfer protocols, and the absence of payer or policy-driven mandates may also contribute. Additionally, fee-for-service reimbursement structures may offer limited support for collaborative, cross-institutional efforts. While our study draws on the broader concept of ECSCs to define regional utilization patterns, OHCA is the focal point of this analysis.

Post-cardiac arrest syndrome^12^ is recognized as a distinct and complex pathophysiological process that requires coordinated efforts to deliver appropriate and timely post-resuscitation care. Given this, there are clear potential benefits from a highly organized systems-based approach to OHCA that can work to assemble these disparate components. Compared to other time-sensitive cardiovascular emergencies, such as STEMI or ischemic stroke, OHCA is less common, with an estimated incidence of 110 per 100,000 persons annually in the U.S^13^. Despite its lower frequency, OHCA imposes a substantial burden on emergency care systems and hospitals due to its high acuity and resource-intensive management. Even among survivors, post-arrest care requires significant coordination and specialized services, justifying the need for system-level approaches. Furthermore, this care can be complicated by centers that see low volumes, lack standardized training and adherence to evidence-based guidelines, or are missing structural or technological capabilities to deliver high-quality post-resuscitation care. Improved outcomes have been demonstrated when a regionalized strategy incorporates pre-hospital and hospital-based guidelines, communication protocols, standardized training, and other critical interventions^14–16^.

Regionalized approaches exist, but historically the focus when designing emergency care systems has been hospital-centric and the use of arbitrary geopolitical boundaries, which does little to encourage inter-hospital cooperation and coordination for ECSCs like OHCA^17^. Financial incentivization by the Centers for Medicare and Medicaid Services (CMS) is also limited, as accountability lies with the treating hospital, and there is little financial motivation for a regionalized approach to building strategic, population-level systems of care for OHCA. A critical next step is to identify and define regional utilization patterns for ECSC, including OHCA, and to create a population-based emergency system of care that moves away from a single hospital or health system-focused approach.

In our prior work, we have described geographic utilization patterns for five emergency care conditions (cardiac arrest, STEMI, ischemic stroke, sepsis or trauma)^18^. We have demonstrated that empirically derived regions can be identified for ECSCs using claims data and that distinct utilization patterns exist from this framework. The objective for this study was to examine if there are regional differences in OHCA outcomes, specifically survival to discharge, utilizing our risk adjustment framework.

## Results

**Fig. 1:**
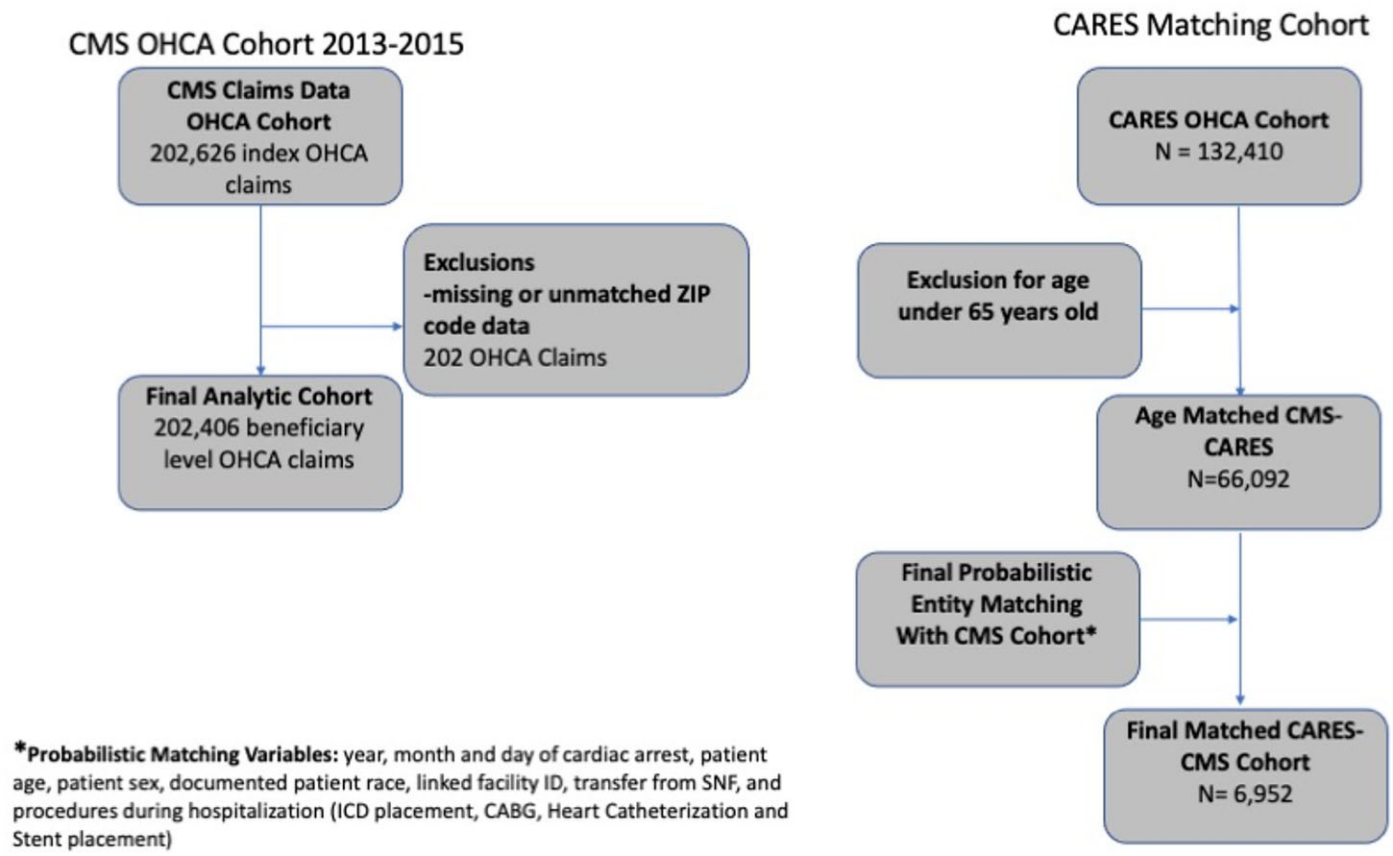
CMS OHCA Cohort and CARES Matching Cohort Flow Diagram. For the CMS cohort Age-eligible ( ≥ 65 years old) beneficiaries were included if they had a valid residential ZIP-code documented on the OHCA claim. For the CARES cohort, all OHCA patients over 65 years old were included in the initial cohort. These methods are further described in Text Boxes 1 and 2, Fig. 4.

After exclusions, 202,406 unique beneficiary-level OHCA claims were included for the final analysis (Fig. 1). Female beneficiaries accounted for 44.5% (*n* = 89,982) of the cohort, 16.2% (*n* = 32,817) were Black beneficiaries, 77.6% (*n* = 157,035) were White beneficiaries, and 6.2% (*n* = 12,554) were Other race or ethnicity beneficiaries (Table 1). Patients aged 65–74 made up 38.2% (*n* = 77,228) of beneficiaries and 50.7% (*n* = 15,250) of survivors. Beneficiaries with five or more comorbidities comprised 21.5% (*n* = 43,563) of the total cohort and 50.2% (*n* = 15,122) of survivors. Overall, 14.9% (*n* = 30,099) survived to discharge.

**Table 1:** CMS OHCA Cohort Characteristics

Variable	Category	Overall	Died	Alive	P-Value
Overall (n)		202406	172,307	30,099	
Age category n (%)	65-74	77,228 (38.2)	61,978 (36.0)	15,250 (50.7)	<0.01
	75-84	72,289 (35.7)	62,074 (36.0)	10,215 (33.9)	
	85+	52,889 (26.1)	48,255 (28.0)	4634 (15.4)	
Sex n (%)	Female	89,982 (44.5)	77,910 (45.2)	12,072 (40.1)	<0.01
	Male	112,424 (55.5)	94,397 (54.8)	18,027 (59.9)	
Race and Ethnicity n (%)	White	157,035 (77.6)	133,261 (77.3)	23,774 (79.0)	<0.01
	Black/African American	32,817 (16.2)	28,231 (16.4)	4586 (15.2)	
	Other*	12,554 (6.2)	10,815 (6.3)	1739 (5.8)	
Comorbidity Count n (%)	0	64,295 (31.8)	62,259 (36.1)	2036 (6.8)	<0.01
	1-2	56,630 (28.0)	51,450 (29.9)	5180 (17.2)	
	3-4	37,918 (18.7)	30,157 (17.5)	7761 (25.8)	
	5+	43,563 (21.5)	28,441 (16.5)	15,122 (50.2)	
Comorbidities					
High Cholesterol n (%)	Yes	42,591 (21.0)	29,320 (17.0)	13,271 (44.1)	<0.01
	No	159,815 (79.0)	142,987 (83.0)	16,828 (55.9)	
Coronary Artery Disease (CAD) n (%)	Yes	35,245 (17.4)	23,040 (13.4)	12,205 (40.5)	<0.01
	No	167,161 (82.6)	149,267 (86.6)	17,894 (59.5)	
Angina n (%)	Yes	917 (0.5)	512 (0.3)	405 (1.3)	<0.01
	No	201,489 (99.5)	171,795 (99.7)	29,694 (98.7)	
Survival	Alive at Discharge n (%)	30,099 (14.9)	–	–	
	Dead	172,307 (85.1)	–	–	

Overall demographic and outcomes characteristics for the CMS Medicare cohort.

*Other: Hispanic; Asian/Native Hawaiian, or Pacific Islander; American Indian or Alaska Native.

Using previously developed regions, which were derived from both OHCA and non-OHCA claims, yielded a total of 205 ECSR for the final analysis. Median OHCA claims per region were 479 (IQR: 113-1,182). The mean population of a region was 1,629,669 ( ± 2,892,298). The mean number of ZCTAs in each region was 175.0 ( ± 174.7). The mean land area of a region was 15,707.7 ( ± 14,872.1) square miles. The mean number of hospitals that treated a patient in the OHCA cohort per region was 67.6 ( ± 72.0).

The final matched cohort consisted of 6592 matching observations from both the CARES registry and CMS datasets. Given the high specificity of our matching process and a lack of hospital identifiers, this resulted in a drop in cohort size from the original age-matched sample of 66,092. For risk adjustment performance, the CARES registry model had an AUC of 0.83. The CMS-CARES matched model had an AUC of 0.72. When applied to the complete CMS cohort, including out-of-sample observations, the AUC was 0.78. The optimal threshold of predicted probability of survival was 0.17.

**Fig. 2:**
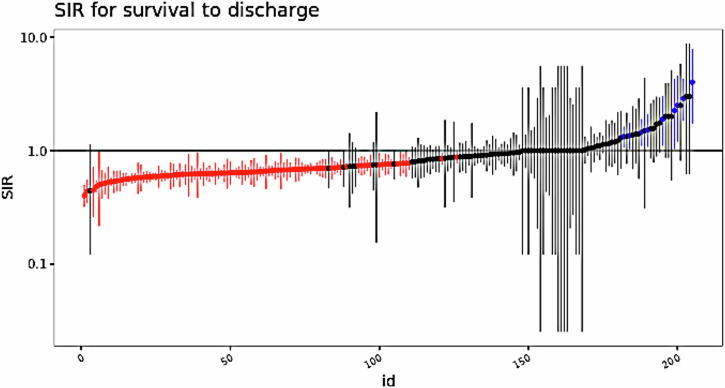
Standardized Incidence Ratio for the outcome of survival to discharge. Caterpillar plot for calculated SIR for each region. Each vertical line represents 95% CI. Red means CI does not cross 1 (underperforming) and blue (overperforming) gray crosses 1 and not statistically significant (95% CI).

Out of the 205 hospital regions, 101 significantly underperformed survival predictions (SIR < 1), while nine overperformed (SIR > 1) (Table 2 and Fig. 2) Mean SIR was 0.91 ± 0.48 with 50% (103/205) regions performing worse than expected and only 4.0% (9/205) of regions performing statistically better than expected (Fig. 2). SIR’s ranged from 0.40 to 4.00. Regional SIR values were significantly clustered with a Moran’s I statistic of 0.14 and a p-value of 0.002. Wider confidence intervals for regions near SIR = 1.0 may reflect statistical instability from small sample sizes or low expected survival counts. These regions reflect low OHCA volumes and should be interpreted cautiously.

**Table 2:** Results by Hospital Region for OHCA Standardized Incidence Ratio

Variable	Category	All Regions	Underperforming Regions (SIR < 1)	Overperforming Regions (SIR > 1)	p-value
Hospital Regions		205	101	9	
Hospitals by Bed Size n (%)					<0.01
	0-99 beds	2229 (48.4%)	997 (47.0%)	123 (32.4%)	
	100-399 beds	1763 (38.3%)	824 (38.9%)	182 (47.9%)	
	400+ beds	453 (9.8%)	221 (10.4%)	61 (16.1%)	
	Unknown	157 (3.4%)	76 (3.6%)	14 (3.7%)	
Hospital Teaching Status n (%)					0.01
	Major Teaching	240 (5.2%)	116 (5.5%)	35 (9.2%)	
	Minor Teaching	4205 (91.4%)	1926 (90.9%)	331 (87.1%)	
	Non-Teaching	157 (3.4%)	76 (3.6%)	14 (3.7%)	
Cardiac Catheterization Lab Availability n (%)					1.00
	Yes	1543 (33.5%)	737 (34.8%)	136 (35.8%)	
	No	2191 (47.6%)	929 (43.9%)	170 (44.7%)	
	Unknown	868 (18.9%)	452 (21.3%)	74 (19.5%)	
Total Population by Region					
	Mean Population (SD)	1,606,308 (2,817,857)	2,880,034 (3,561,705)	220,054 (171,296)	
	Population 65+ years old (SD) (mean %)	227,326 (388,775) (14.2%)	402,641 (490,174) (14.0%)	37,506 (27,387) (17.0%)	
	Male (SD) (mean %)	790,482 (1,379,082) (49.2%)	1,416,129 (1,741,787) (49.2%)	110,101 (83,653) (50.0%)	
	Female (SD) (mean %)	815,825 (1,438,989) (50.8%)	1,463,905 (1,820,239) (50.8%)	109,953 (87,713) (50.0%)	
	White Population (SD) (mean %)	1,184,694 (1,863,053) (73.8%)	2,100,579 (2,292,407) (72.9%)	184,657 (128,066) (83.9%)	
	Black/African American Population (SD) (mean %)	201,243 (433,490) (12.5%)	365,917 (561,711) (17.3%)	23,705 (65,150) (10.8%)	
	Asian Population (SD) (mean %)	80,010 (292,842) (5%)	156,865 (403,758) (5.4%)	1867 (1347) (0.8%)	
	North American Native Population (SD) (mean %)	13,393 (28,769) (0.8%)	19,948 (35,574) (0.7%)	3,063 (4,566) (1.4%)	
	Other Race Population (SD) (mean %)	75,578 (289,251) (4.7%)	143,755 (399,455) (5.0%)	3,189 (3,903) (1.4%)	
	Hispanic Population (SD) (mean %)	286,240 (875,370) (17.8%)	522,935 (1,152,120) (18.2%)	12,721 (12,370) (5.8%)	
Weighted Population by Region					
	Total ZCTA Count (n)	33,120	25,719	823	
	% Below Poverty Weighted mean (Weighted SD)	12.2% (9.2%)	11.9% (8.6%)	12.3% (8.3%)	
	% No Insurance Weighted mean (Weighted SD)	12.9% (7.3%)	13.1% (7.3%)	12.8% (6.9%)	
	% Public Insurance Weighted mean (Weighted SD)	32.4%(11.1%)	31.9% (10.8%)	34.9% (9.2%)	
	% Black Population Weighted mean (Weighted SD)	0.04% (1.1%)	0.01% (0.1%)	0.1% (0.9%)	
	% Unemployed Weighted mean (Weighted SD)	5.3% (2.3%)	5.4% (2.2%)	3.9% (2.2%)	
	% HS Education or More Weighted mean (Weighted SD)	21.1% (5.3%)	21.0% (5.3%)	24.3% (5.0%)	
Weighted Households by Regions					
	Total ZCTA Count (n)	32,989	25,719	823	
	Median Household Income Weighted mean (Weighted SD)	$58,266.41 ($23,687.70)	$59,258.89 ($24,220.54)	$46,429.95 ($11,509.73)	
	% Household Occupancy > 1.5 persons Weighted mean (Weighted SD)	1.0% (1.7%)	1.0% (1.8%)	0.6% (1.3%)	

For unweighted population-level comparisons, we used two-sample t-tests, and for weighted population-level comparisons, we applied weighted t-tests.

### Logistic Regression Model Comparing Overperforming and Underperforming Regions

**Fig. 3:**
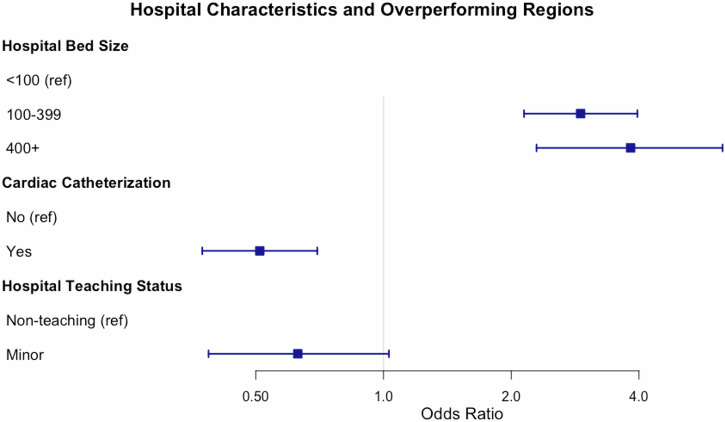
Multiple Logistic Regression Model Overperforming and Underperforming Regions. Multiple logistic regression to test the association of regional performance (overperforming vs. underperforming regions) controlling for hospital size, teaching status, and cardiac catheterization capabilities.

In the multiple logistic regression model testing the association between regional performance and hospital size, teaching status, and cardiac catheterization capabilities we found compared to hospitals with fewer than 100 beds, those with 100–399 beds had 2.91 times higher odds of being in an overperforming region (95% CI: 2.14–3.96), and those with 400 or more beds had 3.82 times higher odds (95% CI: 2.29–6.29) (Fig. 3). Hospitals with cardiac catheterization capability had lower odds of being in overperforming regions (OR: 0.51; 95% CI: 0.37–0.70). Minor teaching hospitals also showed reduced odds (OR: 0.63; 95% CI: 0.39–1.03), though the confidence interval included 1. These results suggest that larger hospital size is positively associated with regional overperformance, while catheterization capability may be inversely associated.

The mean population of an overperforming region was lower than that of an underperforming region (220,054 vs. 2,880,034). The mean population per region was 1,606,308 overall, 2,880,034 in underperforming regions, and 220,054 in overperforming regions. The population aged greater than 65 years old comprised 14.2% overall, 14.0% in underperforming regions, and 17.0% in overperforming regions. Racial and ethnic differences were also present between underperforming and overperforming regions. Racial demographics showed that White individuals made up 73.8% (*n* = 1,184,694) of the population overall, 72.9% (*n* = 2,100,579) in underperforming regions, and 83.9% (*n* = 184,657) in overperforming regions. Black/African American individuals comprised 12.5% (*n* = 201,243) overall, 17.3% (*n* = 365,917) in underperforming regions, and 10.8% (*n* = 23,705) in overperforming regions. Hispanic individuals represented 17.8% (*n* = 286,240) overall, 18.2% (*n* = 522,935) in underperforming regions, and 5.8% (*n* = 12,721) in overperforming regions.

Examining hospital level characteristics, hospitals in overperforming regions had larger capacities (10.4% vs. 16.1% of hospitals with bed size >= 400, *p* < 0.001). For hospital teaching status, across all regions, 5.2% (*n* = 240) were major teaching hospitals and 91.4% (*n* = 4205) were minor teaching hospitals. In underperforming regions, were 5.5% (*n* = 116) and 90.9% (*n* = 1926), respectively. Overperforming regions had 9.2% (*n* = 35) major teaching and 87.1% (*n* = 331) minor teaching hospitals (*p* = 0.007). Cardiac catheterization lab availability was present in 33.5% (*n* = 1543) of hospitals across all regions, 34.8% (*n* = 737) in underperforming regions, and 35.8% (*n* = 136) in overperforming regions. However, this was not statistically significant between overperforming and underperforming regions.

Regarding demographic indicators for weighted results, the overall percentage of Black residents was 0.04% ( ± 1.1) but was greater in overperforming regions (0.1%±0.9) than in underperforming regions (0.01% ±0.10). Overperforming regions also had lower unemployment rates (3.9% ±2.2 vs. 5.4% ±2.3) and a higher proportion of individuals completing at least high school (24.3% ±5.0 vs. 21.0% ±5.3). However, median household income was higher in underperforming regions ($59,259 ± $24,221) than in overperforming regions ($46,430 ± $11,510). Finally, households with more than 1.5 occupants were somewhat more common in underperforming regions (1.0% ±1.8) than in overperforming ones (0.6% ±1.3).

Across all regions, the percentage of residents living below the poverty line averaged 12.2% ( ± 9.2), with slightly higher values in overperforming regions (12.3% ±8.3) compared to underperforming regions (11.9% ±8.6). A similar pattern emerged for rates of uninsured, which averaged 12.9% ( ± 7.3) overall; underperforming regions had a modestly higher percentage (13.1% ±7.3) than overperforming ones (12.8% ±6.9). Although overperforming regions had a modestly higher proportion of publicly insured residents (34.9% vs. 31.7%), this was a descriptive observation and not statistically tested. As such, no inference should be drawn regarding a causal or protective relationship.

## Discussion

Utilizing a novel approach of empirically derived hospital regions from Medicare claims data and a risk adjustment model calibrated using CARES registry data, we identified significant regional disparities for survival to discharge rates for OHCA. Across all regions, we identified a mean SIR for survival to hospital discharge of 0.91 ± 0.48, indicating that on average, regions had slightly lower survival rates than expected. We also noted considerable variability in substantial standard deviation among regions (SIRs ranging from 0.40 to 4.00). Some regions clustered near SIR = 1.0 demonstrated disproportionately wide confidence intervals. This likely reflects statistical noise from low case volumes and sparse predicted survival counts. This highlights the challenge of interpreting regional performance in low-volume settings. In the multiple logistic regression model examining hospital-level predictors of regional overperformance, we note that larger hospital bed capacity was strongly associated with higher odds of being in an overperforming region. Compared to hospitals with fewer than 100 beds, those with 100–399 beds had nearly three times the odds of overperformance, and hospitals with 400 or more beds had almost four times the odds. Conversely, hospitals with cardiac catheterization capability had significantly lower odds of being in overperforming regions, suggesting that while such capabilities are critical for post-arrest care, they may not alone drive regional performance. Minor teaching hospitals also showed lower odds of overperformance, though this finding did not reach statistical significance. Collectively, these results indicate that hospital capacity may be a key structural factor in regional OHCA outcomes, while the presence of specific interventions, such as catheterization labs, may not be sufficient on their own to improve performance at the regional level.

By performance metrics, we noted half of the analyzed regions (101 out of 205) significantly underperformed in terms of survival predictions, while only 4.0% (9 regions) overperformed. By population and demographic factors, we noted that overperforming regions tended to have smaller populations (mean 220,054 vs. 2,880,034 in underperforming regions) and a higher proportion of residents aged 65 and older (17.0% vs. 14.0%). Notably, racial and ethnic composition differed between high and low-performing regions, with overperforming areas having a higher proportion of White residents (83.9% vs. 72.9%) and lower proportions of Black or African American residents (10.8% vs. 17.3%) and Hispanic (5.8% vs. 18.2%) residents. By hospital characteristics, we noted that regions with better OHCA outcomes were associated with a higher proportion of larger hospitals (400+ beds) and major teaching hospitals. These findings highlight the complex interplay of demographic, socioeconomic, and healthcare system factors influencing OHCA outcomes. The marked regional disparities underscore the need for a multifaceted approach to improving OHCA survival rates across the nation through regionalized care approaches, further aligning with proposed AHA strategies.

A comprehensive body of research has previously identified significant regional variation in OHCA survival and outcomes. A landmark study by Nichol et al. from 2008 using the Resuscitation Outcomes Consortium (ROC) Epistry-Cardiac Arrest dataset from 8 US and 3 Canadian sites found significant variation in survival to discharge, ranging from 3.0 to 16.2%^13^. The study further noted substantial disparities in survival rates, OHCA incidence, bystander CPR rates, and EMS response times across regions. Even after adjusting for patient and clinical factors, these site-to-site variations persisted, highlighting the complex interplay of factors influencing OHCA outcomes. In a more contemporary study from 2016, Girotra et al., using the Cardiac Arrest Registry to Enhance Survival (CARES) database, analyzed regional variations in OHCA outcomes across the United States that included 132 U.S. counties from 2011 to 20143. Their findings demonstrated significant disparities in county-level survival rates to discharge, ranging from 3.4% to 22.0%. This nearly sevenfold difference in survival rates across counties was striking and persisted even after adjusting for patient and community characteristics. There were also significant variations in rates of bystander CPR and AED use, ranging from 10% to 64% and 2% to 15%, respectively, across counties. Importantly, Girotra et al. identified that county-level factors accounted for 40.4% of the observed variation in survival, highlighting the crucial role of local and regional factors in determining OHCA outcomes. These factors included not only differences in emergency medical services but also broader community characteristics, such as population demographics, education levels, and socioeconomic status. This study further emphasized the importance of understanding local and regional factors that contribute to OHCA outcomes and suggested that targeted interventions at the county level could potentially improve survival rates.

Our examination of weighted socioeconomic metrics underscores additional nuances. While underperforming regions had marginally lower poverty rates (11.86 vs. 12.31%) and a slightly higher median household income ($59,259 USD vs. $46,430 USD), overperforming regions had a higher proportion of publicly insured residents (34.9 vs. 31.9%), lower unemployment (3.9 vs. 5.4%), and a greater share of individuals with at least a high-school education (24.3 vs. 21.0%). These patterns suggest that income alone may not fully account for differences in OHCA outcomes, and that public insurance coverage, employment, and educational attainment could be important drivers of regional performance.

In the context of this prior research, our study provides a novel and important contribution to understanding regional variations in OHCA outcomes. Using SIRs derived from Medicare claims data and a risk-adjustment model based on registry data, we offer a more comprehensive analysis of regional disparities in OHCA survival that captures all US geographies. We analyzed 202,406 unique beneficiary-level OHCA claims across 205 empirically derived hospital regions, finding SIRs ranging from 0.40 to 4.00. This suggests that regional variations in OHCA outcomes may be even more pronounced than previously recognized, especially when focusing on the Medicare population. This variability in SIRs underscores the critical need for targeted interventions and policy changes to address these persistent disparities. Notably, 49.3% of regions (101 out of 205) significantly underperformed in survival predictions, while only 4.4% (9 regions) overperformed, highlighting the need to further explore the factors contributing to these differences. Furthermore, our study’s focus on Medicare beneficiaries provides crucial insights into OHCA outcomes among older adults, a more vulnerable population that is often underrepresented in prior research. By examining hospital characteristics, such as bed size and teaching status in relation to OHCA outcomes, our study also provides insight into the role of hospital-level factors in regional variations, an aspect not fully explored in previous research. We found that overperforming regions were associated with a higher proportion of larger hospitals (400+ beds) and major teaching hospitals, suggesting that access and timing to larger health systems and hospitals that can provide specialized care may play a crucial role in OHCA survival rates. Cardiac catheterization lab availability however did not show significant differences between overperforming and underperforming regions (*p* = 0.996). This suggests that while cardiac catheterization is a key intervention for specific post-arrest patients, its availability may not be the primary driver of regional differences in overall OHCA survival rates among Medicare beneficiaries. Additionally, our analysis of demographic factors revealed important disparities. Overperforming regions had a higher proportion of White residents (83.9 vs. 72.9% in underperforming regions) and lower proportions of Black/African American (10.8 vs. 17.3%) and Hispanic (5.8 vs. 18.2%) residents. These findings align with and extend previous research on racial and ethnic disparities in OHCA outcomes, emphasizing the need for targeted interventions in diverse communities.

A growing body of evidence supports the concept of CRCs, where specialized post–cardiac arrest care is coordinated regionally. Several studies and a meta-analysis have reported associations between CRC treatment and improved survival or neurological outcomes^19^. The AHA similarly endorses the benefits of CRCs for OHCA, noting that both direct transport and early interfacility transfer to specialized centers can reduce mortality and enhance neurological recovery^20,21^. Our results—demonstrating regions with higher-performing hospitals and more robust care structures tend to have better survival—reinforce calls for regionalization efforts that build on the CRC model. Fostering collaborative networks, promoting interfacility transfer when appropriate, and aligning financial or regulatory incentives could drive improvements in OHCA care at the regional and national levels.

Our study provides a comprehensive, nationwide assessment of OHCA outcomes among Medicare beneficiaries, offering valuable insights into regional disparities, demographic factors, and hospital characteristics that influence survival rates. Unlike prior studies that relied on registry data from selected hospital systems, our study leverages nationally representative Medicare claims data to examine risk-adjusted OHCA outcomes across empirically derived regions—offering a novel, more generalizable approach to evaluating geographic disparities in survival. These findings can inform policy decisions, resource allocation, and future research aimed at improving OHCA outcomes and reducing disparities across different regions in the United States.

This study’s findings have several important limitations. Medicare administrative claims data provide a broad national perspective but lack the sensitivity and specificity of registry data for identifying OHCA cases. Our findings are limited to Medicare beneficiaries aged 65 and older, limiting generalizability to younger populations with potentially different risk factors and outcomes. Patients included in our analysis must survive to reach the emergency department, possibly inflating our reported survival rate (14.9%) compared to registry cohorts. Furthermore, the predominantly White beneficiary composition of our sample (77.6%) restricts generalizability to more diverse populations. Administrative claims also lack detailed clinical and pre-hospital information, preventing deeper insights into care quality and specific interventions. Our hospital region definitions, though empirically derived, may not exactly match real-world care delivery patterns for OHCA. While the study relies on data from 2013–2015, national guidelines regarding OHCA regionalization have remained constant, thus preserving the relevance of our findings to current system planning. Additionally, due to constraints imposed by probabilistic matching without hospital identifiers, only a subset of registry data could be confidently linked to CMS claims. This necessary exclusion of unmatched encounters may limit the representativeness of the matched cohort and introduce potential selection bias. Despite these limitations, this study’s nationwide scope and large Medicare cohort offer valuable insights into regional variability in OHCA outcomes and highlight potential disparities. Using Medicare claims data in the absence of a comprehensive national OHCA registry remains essential for understanding regional variations in survival.

In conclusion, our study provides a comprehensive nationwide assessment of OHCA outcomes among Medicare beneficiaries, offering valuable insights into regional disparities, demographic factors, and hospital characteristics that influence survival rates. Our findings reveal significant variations in OHCA outcomes across the United States, underscoring the critical need for targeted interventions and policy changes to address these persistent disparities. Moving forward, a multifaceted approach that addresses pre-hospital care, hospital capabilities, and community-level factors will be crucial to narrowing the gap in OHCA outcomes and improving patient outcomes.

## Methods

Using Medicare Fee-For-Service (FFS) claims, we identified beneficiaries receiving care for OHCA in the Medicare Provider Analysis and Review (MEDPAR) and/or Outpatient FFS Research Identifiable Files (RIF) from 2013 to 2015. These years of data were used because of the available access to the full dataset to the study team. OHCA beneficiaries were identified using ICD-9-CM codes 427.5, 427.41, 427.42 and mapped ICD-10-CM codes I46, I49.01, I49.02 (International Classification of Diseases, Ninth and Tenth Revision, Clinical Modification) as the primary or admitting diagnosis and based on previously published methods for identifying OHCA patients^22–25^. Age-eligible ( ≥ 65 years old) beneficiaries were included if they had a valid residential ZIP-code documented on the OHCA claim.

CMS RIF data includes claim-level information for each encounter, including discharge diagnosis codes, patient age at encounter, sex, and race/ethnicity as a single category. Comorbidity scores were calculated for all patients based on diagnosis codes listed in the claim utilizing the “icd” package in R version 4.0.3 (R Foundation for Statistical Computing, Vienna, Austria, 2020)^26^. The CMS Vital Status file, containing beneficiary dates of death through June 2019, was used to calculate survival for patients.

To develop the risk adjustment model, a matched cohort was identified between Medicare claims and the Cardiac Arrest Registry to Enhance Survival (CARES). The matched cohort was used to evaluate the performance of the novel, claims-based, risk-adjustment model against a registry developed risk-adjustment model (Table 3). CARES encounters were excluded if they were missing data on survival to hospital discharge, race and ethnicity, or whether the cardiac arrest was witnessed or not.

**Table 3:** Cardiac Arrest Registry to Enhance Survival (CARES) cohort

Variable	Category	Overall	Died	Alive	P-Value
N (%)		132,410	118,129 (89.2)	14,281 (10.8)	
Age n (%)	< 65	66,092 (50.0)	57,007 (48.3)	9085 (63.6)	<0.01
	65-74	27,714 (20.9)	24,795 (21.0)	2919 (20.4)	
	75-84	23,015 (17.4)	21,388 (18.1)	1627 (11.4)	
	85+	15,490 (11.7)	14,852 (12.6)	639 (4.5)	
	Unknown	99 (0.0)	87 (0.0)	12 (0.1)	
Gender n (%)	Female	51,670 (39.0)	46,746 (39.6)	4924 (34.5)	<0.01
	Male	80,732 (61.0)	71,375 (60.4)	9357 (65.5)	
	Unknown	8 (0.0)	8 (0.0)	0 (0.0)	
Race / Ethnicity n (%)	White	60,863 (46.0)	53,792 (45.5)	7071 (49.6)	<0.01
	Black / African American	26,856 (20.3)	24,537 (20.8)	2319 (16.2)	
	Other	44,691 (33.7)	39,800 (33.7)	4891 (34.2)	
Alive at Discharge n (%)	Alive	118,129 (89.2)	-	-	
	Dead	14,281(10.8)	-	-	

Demographic and outcome characteristics.

In brief, CARES was first established in 2004 in partnership with the Centers for Disease Control and Prevention (CDC) and is a federally supported registry aimed at improving data collection for OHCA^27,28^. Participating centers complete standardized data elements about out-of-hospital cardiac arrest cases, including prehospital and in-hospital variables. The registry follows the Utstein template for data collection in cardiac arrest^29,30^. EMS agencies submit demographic, cardiac arrest, and treatment data to the registry for all EMS-treated cardiac arrests, and the receiving hospital submits hospital-based treatment and outcome data. Currently, more than 2300 EMS agencies and 2500 hospitals participate in CARES data collection, representing a catchment area of approximately 179 million, which includes 34 statewide registries.

Record matching between CMS and CARES datasets was performed using a two-stage process. In the first stage, a probabilistic entity matching was performed to link hospital IDs between the two datasets, as the hospital IDs in the CARES dataset were masked. The matching process involved comparing every group of claims to each other, with each match within the claims data receiving 1 point. Ten variables were used in the probabilistic matching between CARES OHCA events and CMS OHCA claims: year, month, and day of OHCA; patient age; patient sex; documented patient race and/or ethnicity; linked hospital facility ID; transfer from a skilled nursing facility (SNF); and procedures that occurred during hospitalization (ICD placement, CABG, cardiac catheterization, and/or PCI). Procedures were identified by procedure code for CMS data and variable field for CARES data. A perfect match would result in a score of 9, while a non-match would result in a score of 0. Once matched pairs were identified, the data were aggregated at the institutional level. In the second stage, matched entities from the first stage were used as a block for the secondary case match, which required matching at the hospital level. In the second stage, only high-probability, non-ambiguous (unique) matches were used to develop the risk adjustment model.

**Fig. 4:**
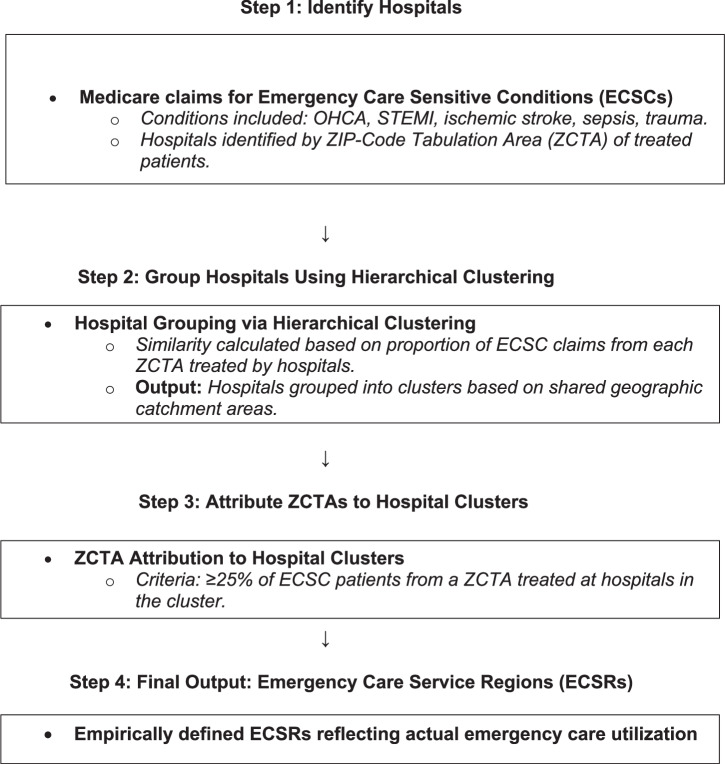
Emergency Care Service Regions (ESCR) Formation Methodology. Stepwise description of the methodology utilized for the development of ECSR regions using Medicare data.

To describe regional trends in cardiac arrest outcomes, we utilized previously defined emergency care service regions (ECSR) (Text Boxs 1 and 2, Fig. 4)^18^. ECSRs represent a geographic area within which a portion of patients seek care at an empirically defined group of hospitals. These regions do not follow standardized health system catchment areas or boundaries and were developed using our previously described methods. We utilized the following approach: ECSRs were formed by identifying groups of hospitals that provide care for beneficiaries with ECSCs (cardiac arrest, STEMI, ischemic stroke, sepsis or trauma) in the same geographic areas as defined by ZIP-Code Tabulation Areas (ZCTAs). Once groups of hospitals were assigned to a region using hierarchical clustering ZCTAs were attributed to these regions if greater than 25% of the patients in those ZCTAs were treated by the hospitals in that region. This method allows for attribution of one ZCTA to more than one region when patient care is distributed.

The primary outcome of interest was survival to hospital discharge by standardized incidence ratio (SIR). This was determined using the CMS Vital status file. If a patient’s date of death was documented as one or more days post-discharge, they were considered alive at hospital discharge. The SIR for survival to discharge was calculated as the proportion of observed survivors divided by the sum of predicted survival counts to hospital discharge, using the risk-adjustment model described below.

Using the linked cohort from the Cardiac Arrest Registry to Enhance Survival (CARES) and Medicare Claims (CMS) data, two logistic regression models were constructed, each predicting survival to hospital discharge with variable selection based on prior literature. The first model utilized only registry data from CARES, while the second model leveraged only claims data from CMS. Both models controlled for age, sex, and race and ethnicity^31^. The registry model also included the following variables: location of cardiac arrest, presence or absence of bystander CPR, whether the arrest was witnessed or not, if the first cardiac rhythm was shockable, if an AED was applied prior to EMS arrival, and if there was sustained ROSC^3,32–35^. For the CMS cohort, we trained a logistic regression model with 30-fold cross-validation utilizing a 70/30 training/test modality. This model controlled for patient age, gender, race and ethnicity, the presence of high cholesterol by ICD-9-CM and ICD-10-CM codes, and Charlson Comorbidity Score^36^. Performance characteristics were calculated for both models and using both in-sample and out-of-sample testing cohorts. The Area Under the Curve (AUC) was calculated for each model to assess how accurately the models could predict survival to hospital discharge. Of note, while more straightforward methods (e.g., stratified analysis or matching) were considered, these approaches do not allow for the same precision or adaptability across a heterogeneous national population. The two-model approach allowed us to build and validate a robust risk adjustment framework with clinical grounding (via the registry model) while ensuring scalability and feasibility for population-level outcomes (via the claims model).

Applying the above claims-based, risk-adjustment model, we calculated the predicted probabilities of survival for each OHCA beneficiary using the full CMS OHCA dataset. The optimal cut point for predicting survival was calculated by maximizing Cohen’s Kappa and implemented using the cutpointr function^37^. The regional SIR was calculated as the ratio of the observed survivors to the predicted survivors with a probability greater than the optimal cutpoint. Confidence intervals were calculated using the method described by Morris and Gardner^38^. Additionally, spatial autocorrelation of region SIR was evaluated using a Monte Carlo simulation of Moran’s I to evaluate clustering and dispersion of values. To ensure the robustness of our results, we also performed multiple logistic regression to test the association of regional performance (overperforming vs. underperforming regions) controlling for hospital size, teaching status, and cardiac catheterization capabilities. The model predicted the likelihood of a hospital being in an overperforming region. Because of data imbalance and potential for nonconvergence of our models, we leveraged the full regional hospital dataset to include a comparison of 4,265 hospitals to categorize overperforming or underperforming regions (380 hospitals in overperforming regions and 3,885 in underperforming regions). To place the results into context, we utilized 2015 ACS 5-Year Estimate ZCTA-level data and calculated the following variables: mean percentage Below Poverty, percentage Black population, percentage no Insurance, percentage public Insurance, percentage unemployed, and percentage high School education or more. This was weighted on total population within ZCTA’s for ZCTA’s in both over and underperforming regions. All means were weighted on the total number of households for median household income (HHI) and the percentage of households with occupancy > 1.5 persons. This weighting was performed to ensure that each region’s contribution reflects its population size (or total households), rather than simply averaging across ZCTAs. All analysis were conducted in R version 4.0.3 (R Foundation for Statistical Computing, Vienna, Austria, 2020). The study followed STROBE guidelines^39^ (Supplementary Table 1) and was approved by the Institutional Review Board at the Icahn School of Medicine at Mount Sinai (STUDY-20-00366).

Box 1 Stepwise Summary of Risk Adjustment Methodology

1. Data Sources
  1. Medicare CMS Data (2013–2015): Identified OHCA encounters from MEDPAR and Outpatient claims using ICD-9/10-CM codes for cardiac arrest. Additional demographic and comorbidity (Charlson) data were retrieved from administrative records. Vital status determined from CMS files.
  2. CARES Registry Data: Provided detailed variables (e.g., location of arrest, bystander CPR, first rhythm) following the Utstein template.
2. Record Matching
  1. Hospital ID Linkage: CARES hospital IDs are masked. We performed probabilistic match using 10 variables (e.g., date of arrest, age, sex, race/ethnicity, certain procedures) to align hospital encounters between CARES and CMS. Hospital-level identifiers were not provided for CARES data.
  2. Patient-Level Match: For high-probability matches, each CARES event was paired with its CMS claim. Ultimately, 6,592 matched observations were obtained.
3. Risk Model Development
  1. CARES Registry Model: Logistic regression predicting survival to hospital discharge using CARES variables:
    1. Age
    2. Sex
    3. Race/Ethnicity
    4. Location of arrest (public, private)
    5. Bystander CPR (yes/no)
    6. Witnessed vs. unwitnessed
    7. Shockable rhythm (VF/VT)
    8. AED applied prior to EMS arrival
    9. Sustained ROSC
  2. CMS Claims Model: Separate logistic regression predicting survival, trained only on claims-based variables available in Medicare:
    1. Age
    2. Sex
    3. Race/Ethnicity
    4. High cholesterol (ICD code)
    5. Charlson Comorbidity Score
4. Model Validation
  1. Both models were evaluated via Area Under the Curve (AUC).
  2. CMS model was 30-fold cross-validated (70/30 split). Final AUC in out-of-sample testing was 0.776.
5. Calculating Standardized Incidence Ratios (SIR)
  1. For each OHCA claim in full CMS dataset, final CMS model generated predicted probability of survival.
  2. Individuals with probabilities >0.171 (optimal cutpoint by Cohen's Kappa) were labeled "predicted survivors."
  3. At region level, SIR = (Observed survivors)/(Sum of predicted survivors).
  4. Confidence intervals calculated following Morris and Gardner's method.

Box 2 Formation of Emergency Care Service Regions (ECSRs) Hospitals treating Medicare beneficiaries for emergency care sensitive conditions (ECSCs)—out-of-hospital cardiac arrest (OHCA), STEMI, ischemic stroke, sepsis, and trauma—were first identified from CMS claims data, based on the ZIP Code Tabulation Areas (ZCTAs) of patient primary residence listed on each claim. Hospitals were subsequently grouped into clusters using agglomerative hierarchical clustering, which calculates similarity based on each hospital’s distribution of ECSC patients across ZCTAs. This clustering produced candidate hospital regions sharing geographic catchment areas for emergency care. Finally, ZCTAs were attributed to these hospital clusters to define ECSRs. Attribution was based on the criterion that at least 25% of ECSC patients from a ZCTA received treatment at hospitals within a given cluster. This threshold allowed overlapping attribution, where a single ZCTA could be assigned to multiple ECSRs if multiple hospital clusters each treated ≥25% of the ZCTA's ECSC cases. This methodology resulted in empirically derived ECSRs reflecting true utilization patterns of emergency care, rather than predefined health system or geopolitical boundaries, and served as the basis for subsequent analyses of regional OHCA outcomes.

## Supplementary information

Supplementary Information

## Data Availability

The Medicare data used in this study are Research Identifiable Files (RIFs) and are not publicly available. These datasets can be requested by researchers through the Centers for Medicare & Medicaid Services via the Research Data Assistance Center (ResDAC) (https://www.resdac.org). While CMS provides publicly available summary data (https://data.cms.gov), these do not contain the individual-level, geographic, or clinical detail needed to replicate our findings. CARES registry data are also restricted and may be requested via https://mycares.net. Access to both datasets require institutional approval and data use agreements.
