# Supplementary material for: Population-based risk adjusted outcomes for out-of-hospital cardiac arrest
Source: NPJ Cardiovasc Health. 2026 Mar 2;3:8. doi: 10.1038/s44325-026-00108-7 (PMC12953574; doi:10.1038/s44325-026-00108-7)
Supplement: Supplementary file 1 — Supplementary Information [file 44325_2026_108_MOESM1_ESM.pdf]

Supplementary Files for Population-based Risk Adjusted Outcomes for Out-of-hospital Cardiac Arrest by Abbott et al.

1. Supplementary Figure 1: Map of SIR distributions for regions of the US.
2. Supplementary Figure 2: Map of lower non-significant SIR values for regions of the US
3. Supplementary Table 1: STROBE Checklist

Supplementary Figure 1: Map of SIR distributions for regions of the US.

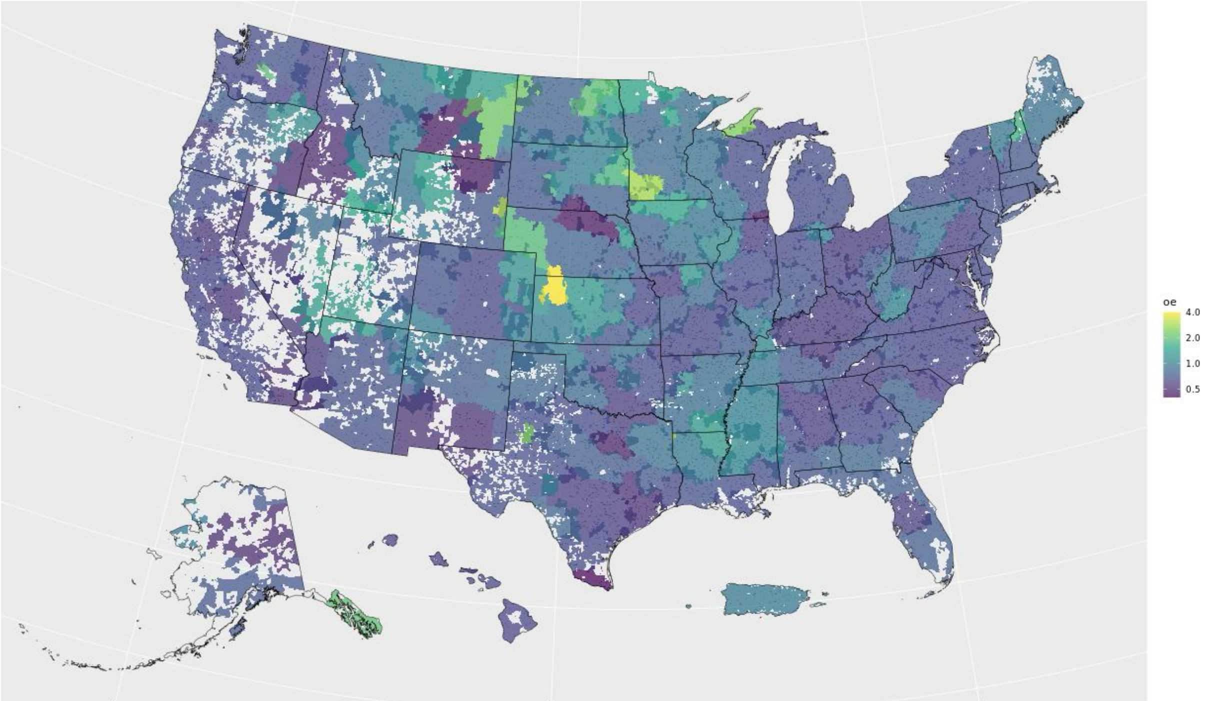

Supplementary Figure 2: Map of lower non-significant SIR values for regions of the US. Legend: Red – Underperforming, Blue – Overperforming, Grey – Non-significant, White - No data available.

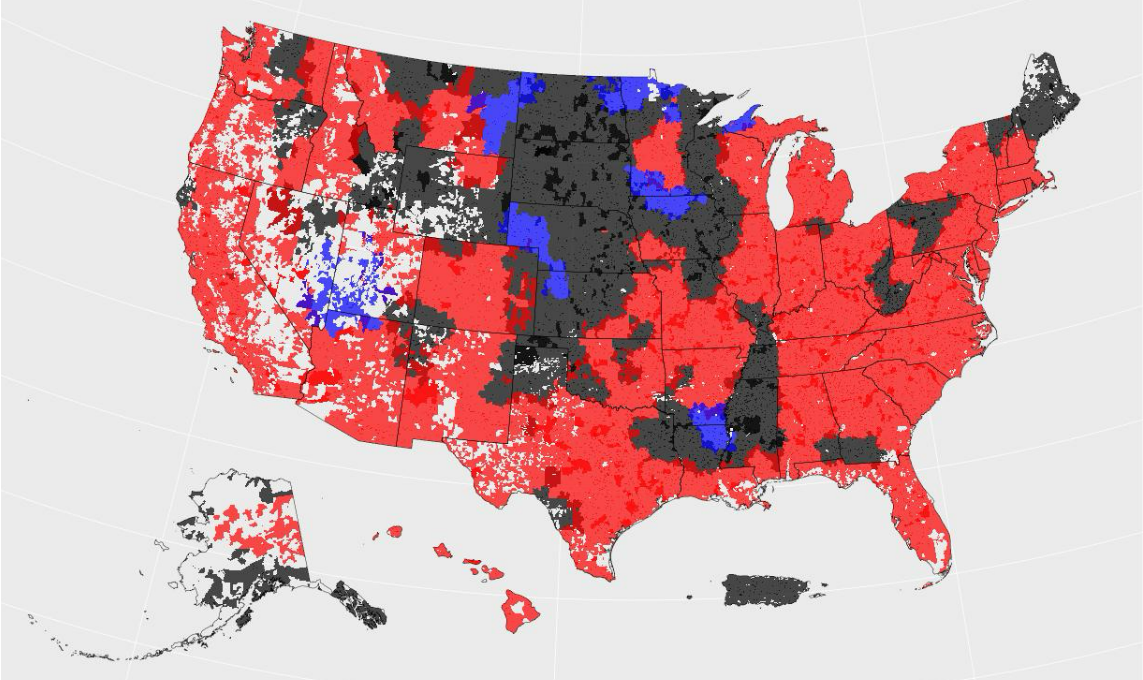

Supplementary Table 1: STROBE Checklist for Cohort Studies

Section	Item No		Recommendation / How Addressed
Title and abstract	1(a)		Study design ('population-based risk adjustment model') mentioned in title and abstract.
	1(b)		Abstract provides a summary including background, methods, and key findings.
Introduction		2	Scientific rationale provided in Introduction, citing regional OHCA survival variation and need for system-level care.
		3	Objective: to examine regional variation in OHCA outcomes using a risk adjustment framework.
Methods		4	Study design: retrospective cohort using Medicare and CARES data.
		5	Setting: U.S. Medicare population, 2013-2015; data sources and timelines detailed in Methods.
	6(a)		Eligibility: Medicare FFS , over 65 years old, OHCA ICD codes; matched CARES cohort described.
	6(b)		Matched cohort described with selection criteria, but no traditional 'exposed/unexposed' groups.
		7	Outcomes: survival to discharge; predictors: demographics, comorbidities, OHCA-level features; variables defined in Methods and Supplemental A.
		8	Data sources: CMS and CARES; measurement methods detailed for each variable including risk modeling.
		9	Bias addressed through model calibration, cross-validation, and use of external registry data.
		10	Study size based on all eligible CMS OHCA claims from 2013-2015 (n=202,406).
		11	Quantitative variables modeled using logistic regression; Comorbidity scores and other continuous measures treated appropriately.
	12(a)		Logistic regression models with cross-validation as described; confounders adjusted in models.
	12(b)		Subgroup analysis via logistic regression comparing over/underperforming regions.
	12(c)		Missing data addressed via exclusion (e.g., incomplete registry records).
	12(d)		No traditional follow-up; outcome assessed at discharge.
	12(e)		Sensitivity tested via model validation and spatial autocorrelation.
Results	13(a)		Flow diagram in Figure 1; n=202,406 included in final cohort.
	13(b)		Exclusions and reasons described in text.
	13(c)		Flow diagram used.
	14(a)		Descriptive characteristics shown in Table 1 and Table 2.
	14(b)		Missing data noted in CARES exclusions.
	14(c)		No long-term follow-up: endpoint is survival to hospital discharge.
		15	Outcome (survival to discharge) presented overall and by subgroup.
	16(a)		Unadjusted and adjusted ORs provided in multiple regression analyses.
	16(b)		Continuous variables like hospital size categorized; boundaries noted.

	16(c)	Absolute risks not calculated as focus is on SIRs and ORs.
	17	Subgroup and sensitivity analyses reported (e.g., regional performance predictors).
Discussion	18	Key findings summarized in relation to objectives.
	19	Limitations discussed in detail (e.g., generalizability, administrative data).
	20	Balanced interpretation acknowledging limitations and comparison to prior work.
	21	Generalizability addressed, especially for Medicare population.
Other information	22	Funding sources and roles detailed in funding section.
